# Construction of a demand and capacity model for intensive care and hospital ward beds, and mortality from COVID-19

**DOI:** 10.1186/s12911-021-01504-y

**Published:** 2021-04-27

**Authors:** Christopher Martin, Stuart McDonald, Steve Bale, Michiel Luteijn, Rahul Sarkar

**Affiliations:** 1 Director of Modelling At Crystallise, Unit 19, Saffron Court, Southfields Business Park, Basildon, Essex SS15 6SS UK; 2 Honorary Researcher, University College London, London, UK; 3 Head of Demographic Assumptions and Methodology at Lloyds Banking Group, 25 Gresham Street, London, EC2V 7HN UK; 4 Senior Actuary at Munich Re UK Life Branch, 10 Fenchurch Avenue, London, EC3M 5BN UK; 5 Biometric Research Data Specialist at Hannover Re UK Life Branch, 10 Fenchurch Street, London, EC3M 3BE UK; 6 Consultant Physician in Respiratory Medicine and Critical Care at Medway NHS Foundation Trust, Windmill Road, Gillingham, Kent ME7 5NY UK

**Keywords:** COVID-19, Demand, Capacity, Model, Mortality, Public health, Infectious diseases

## Abstract

**Background:**

This paper describes a model for estimating COVID-19 related excess deaths that are a direct consequence of insufficient hospital ward bed and intensive care unit (ICU) capacity.

**Methods:**

Compartmental models were used to estimate deaths under different combinations of ICU and ward care required and received in England up to late April 2021. Model parameters were sourced from publicly available government information and organisations collating COVID-19 data. A sub-model was used to estimate the mortality scalars that represent increased mortality due to insufficient ICU or general ward bed capacity. Three illustrative scenarios for admissions numbers, ‘Optimistic’, ‘Middling’ and ‘Pessimistic’, were modelled and compared with the subsequent observations to the 3rd February.

**Results:**

The key output was the demand and capacity model described. There were no excess deaths from a lack of capacity in the ‘Optimistic’ scenario. Several of the ‘Middling’ scenario applications resulted in excess deaths—up to 597 deaths (0.6% increase) with a 20% reduction compared to best estimate ICU capacity. All the ‘Pessimistic’ scenario applications resulted in excess deaths, ranging from 49,178 (17.0% increase) for a 20% increase in ward bed availability, to 103,735 (35.8% increase) for a 20% shortfall in ward bed availability. These scenarios took no account of the emergence of the new, more transmissible, variant of concern (b.1.1.7).

**Conclusions:**

Mortality is increased when hospital demand exceeds available capacity. No excess deaths from breaching capacity would be expected under the ‘Optimistic’ scenario. The ‘Middling’ scenario could result in some excess deaths—up to a 0.7% increase relative to the total number of deaths. The ‘Pessimistic’ scenario would have resulted in significant excess deaths. Our sensitivity analysis indicated a range between 49,178 (17% increase) and 103,735 (35.8% increase). Given the new variant, the pessimistic scenario appeared increasingly likely and could have resulted in a substantial increase in the number of COVID-19 deaths. In the event, it would appear that capacity was not breached at any stage at a national level with no excess deaths. it will remain unclear if minor local capacity breaches resulted in any small number of excess deaths.

**Supplementary Information:**

The online version contains supplementary material available at 10.1186/s12911-021-01504-y.

## Background

The number of COVID-19 deaths in the UK was 74,125 deaths and the number of known cases was 2,542,069 as of the 2nd January 2021 representing a case fatality rate (CFR) of 2.9%. The Office for National Statistics (ONS) Coronavirus (COVID-19) Infection Survey estimated that 8.7% of people in England still had antibodies at detectable levels based upon serological testing [1]. Assuming that, as of the 2nd January 2021, around 10% to 15% of the population of England has been infected (taking into account those with antibodies no longer at detectable levels, the lag time and the small proportion of false negative serology), then this would suggest that between 6.7 million and 10 million people had already been infected representing an infection fatality rate (IFR) of 0.7% to 1.1%.

IFR estimates are typically made in the context of adequate capacity of health care services including hospital beds and ICU beds. Should the demand for ICU beds exceed the supply, then the IFR would be expected to rise. In this paper we describe a demand and capacity model designed to estimate the number of COVID-19 deaths that would directly arise from a lack of ICU and ward beds. Such a model currently does not exist for the UK in the public domain.

In November 2020 we began development of a model to estimate what increases in deaths could be expected as a direct consequence of a lack of hospital bed capacity, and in particular ICU beds. Due to availability of data, the model was restricted to England.

Estimating the ICU capacity in England is complicated, as the actual capacity at a given point of time varies between usual bedbase and the maximum surge capacity created to handle increasing demand during a pandemic. There were estimated to be 4114 ICU beds in England pre-pandemic [2]. However, there are plans in place to allow surges in ICU capacity when pandemics occur which entails repurposing other hospital resources including anaesthetic rooms, operating theatres and parts of accident and emergency departments. The aim was to be able to increase ICU bed capacity across the country by 100% in the event of a pandemic [3]. It is likely that the increase in capacity will vary by institution and one case study managed to increase capacity by 236% during the first wave [4]. In the event of a pandemic, the NHS is expected to respond through a whole system approach, which has been outlined in the UK CRITCON scoring system. The basis for the system is that the same non-pandemic ethical standards are applied to treat patients and allocate resources, unless in the extreme scenario (CRITCON 4). The system was formulated in 2009 for H1N1 and has been updated in 2020 in the context of the COVID-19 pandemic. The levels described above extend from a normal capacity at CRITCON 0 to CRITCON 4 when the system is overwhelmed. A “mutual aid” system exists to facilitate inter-hospital or regional transfer of patients when the local intensive care capacity is breached in order to support the principle that no patient should be deprived of the appropriate care if there is systemwide capacity available. Eighteen intensive care networks in the England and Northern Ireland manage the capacity and allocation to intensive care in the event of increasing demand.

At the start of the pandemic a series of field hospitals were constructed at eight sites across England. The capacity provided by these field hospitals is flexible according to circumstance, but it is estimated that this would potentially provide an additional 8000 general ward beds and 500 ICU beds though there may be staffing constraints that limit this number [2].

In this paper we describe a model for estimating the number of additional deaths that occur from a lack of capacity under different scenarios. These are illustrative scenarios and not forecasts. Parameterization of the model can be modified according to available information and updated over time.

## Methods

### Model description

The model can be found on GitHub: https://github.com/Crystallize/COVID19_ExceedingCapacityModel (Additional file 1).

The model is a series of static compartmental models that estimates the number of COVID-19 related deaths in England up to late April 2021 under three conditions: availability of both general ward and ICU care; availability of general ward care but no ICU care; and no availability of either general ward or ICU care.

The model was developed as an Excel workbook as Excel allows rapid iteration and development with transparency for other developers and reviewers as it is a widely available platform.

### General outline

**Fig. 1:**
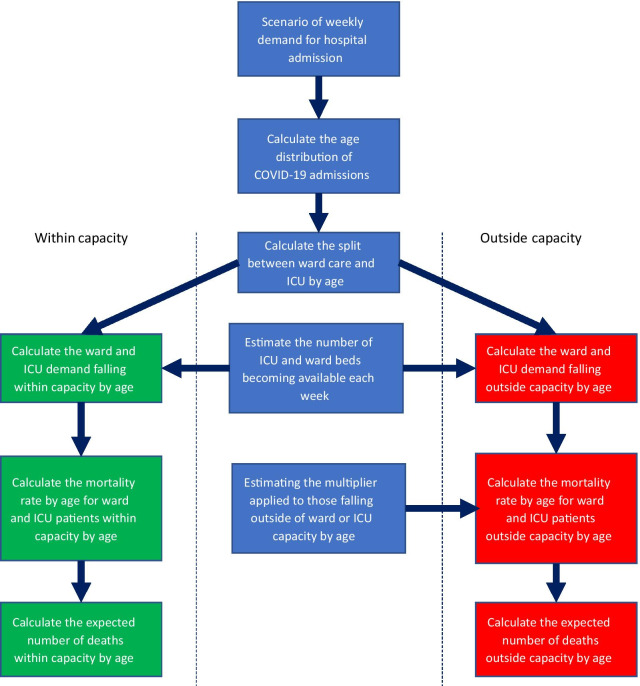
Outline of structure of model

The general outline of the model is shown in Fig. 1 Outline of the structure of the model. It operates using a scenario of COVID-19 hospital admission demand in weekly time steps.

**Fig. 2:**
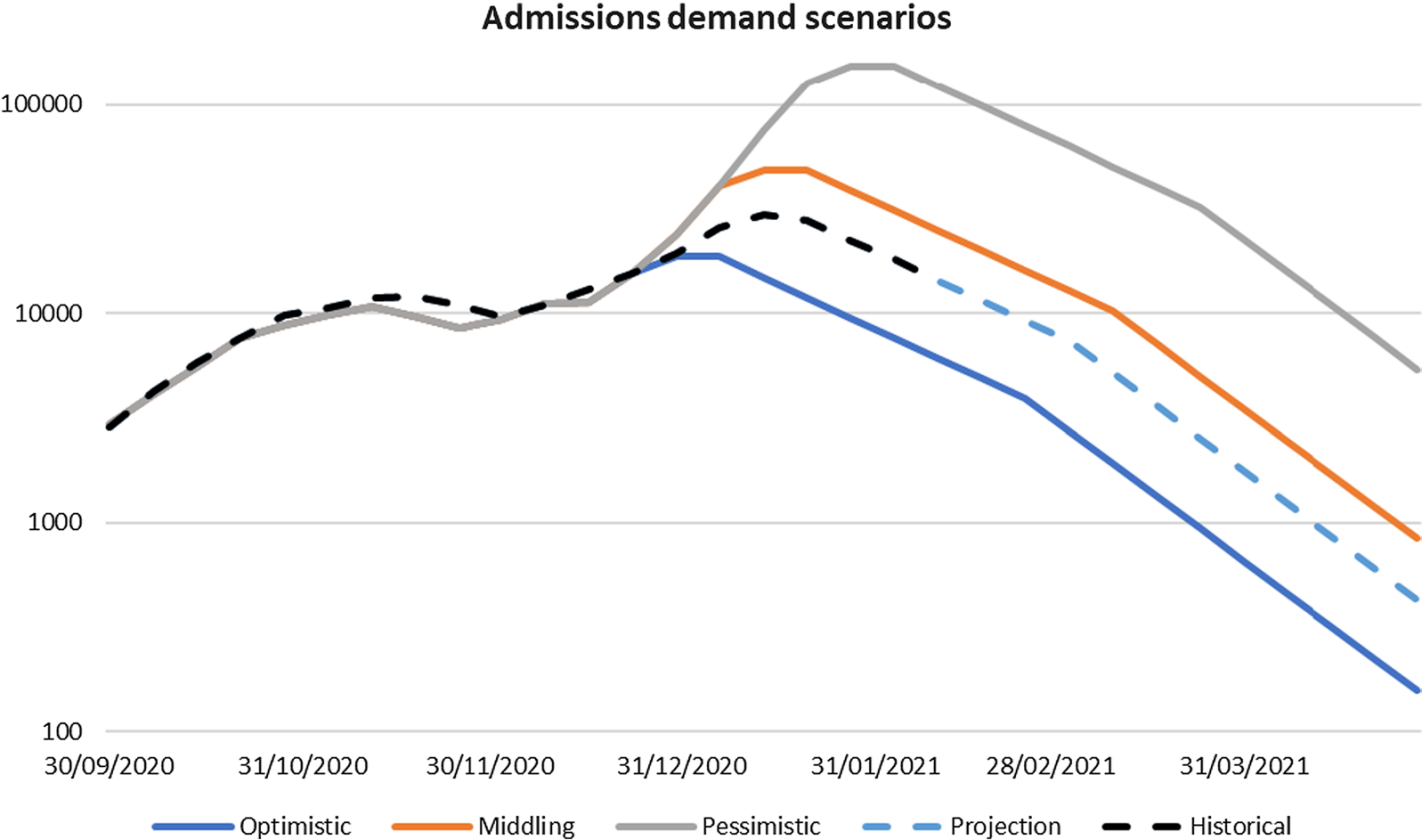
Graph showing three scenarios of projected admission demand from the 9th December 2020”

First, we modelled the expected age distribution of admissions. This is an important input as hospital and intensive care admission rates and COVID-19 mortality vary substantially by age. Subsequently, the weekly demand for intensive care and ward beds was modelled by age. The weekly availability of beds was calculated using estimates of the number of free beds usually available in ICUs in England plus any surge capacity and additional capacity freed by the cancellation of routine surgery, field hospitals and the use of private facilities adjusted for the average duration of stay on ICU and in the ward in general.

We then compared the weekly demand for ward and ICU beds with the maximum number of beds available. This allowed for the number of weekly admissions that fall within or outside of capacity.

Next, we calculated an estimate of the multiplier of the mortality rates when an ICU bed is not available to someone who needs it, and similarly for general ward care. This was done by compartmentalizing ward and ICU patients to categories of care for which estimates were made and then aggregated up to the ward or ICU level again. The assumption is that the ICU mortality rate is multiplied by 1.99 if there is no ICU bed available, but there is a ward bed, and by 9.02 if there is neither a ward nor ICU bed available. For general ward patients, the multiplier is 3.69 if there are no ward beds available. More detail on the derivation of this is provided in the section on the “Excess Mortality Model”. Sensitivity testing indicated that results were not especially sensitive to small changes in these mortality multipliers (± 20%).

Once COVID-19 mortality rates for ward and ICU patients both within and outside of capacity were calibrated, the number of deaths occurring ‘within capacity’ and the number that occur directly as a result of being out of capacity could be calculated. The number of ‘out of capacity’ deaths does not include the number of deaths that would have been expected to occur if normal care had been received, and so represent ‘excess’ deaths occurring because the capacity limit was breached.

Description of each step in the modelling.

We will now describe the purpose and workings of the model in more detail.

### Principle parameters

The pre-pandemic (i.e., before March 2020) spare bed availability in the general wards in England is estimated at 9,769 and the ICU spare bed capacity as 817 beds from a study of hospital capacity in the COVID-19 pandemic by the Medical Research Council, Public Health England, The National Institute of Health Research and Imperial College [2]. The same study estimated an additional 1,810 ICU beds and 52,498 beds could be acquired using field hospitals, the cancellation of routine care and the use of private hospital facilities. In addition, there are pandemic response plans in place to increase ICU capacity with a surge in demand. This surge capacity would be intended to increase ICU beds by 100% by using anaesthetic rooms, operating theatres and other hospital resources [3]. Altogether this would increase spare ICU capacity to 3,444 beds and spare ward capacity to 62,267 beds. These 62,267 ward beds can provide for 87,174 patients weekly. These figures are based on an estimated length of stay on ICU of 7 days and length of stay on the general ward of 5 days [5, 6].

### Estimating COVID-19 admissions by age

The age distribution of admissions was required in 5-year age bands up to the age of 80 years with one category for those of age 80-years and over (if this data is available it can be applied directly in the model removing the need for this estimation step). The age distribution in ICUs is lower than on the wards with an average age of 62 years and with only a small proportion of those over 80-years being fit enough for invasive ventilation [5]. However, we estimated the distribution of admissions for each age-band for each calendar week by fitting an exponential curve to age-binned data on admission rates for adults, then interpolating admission rates for each year of age. Using population estimates from 2019 for each year of age, estimates of the numbers of admissions were made [7]. These were then recalibrated so that the exact number of admissions in each age group matched the observed numbers in each corresponding age-band.

### New COVID-19 patients in hospital

In this step, we applied the distribution of admissions by age to the weekly admission demand from the scenarios to calculate the percentage of admissions accounted for by each year of age.

### Splitting COVID-19 admissions into ICU and ward

Next, we calculated the proportion of admissions that require intensive care and ward care by age for each 5-year age band, with one age band for those aged 80 years or over. This is done using data on the age breakdown of admissions and total admission numbers to ICU from the ICNARC report of the 18^th^ December 2020, and the total number of COVID-19 hospital admissions up to the 18^th^ December 2020 from the Gov.UK COVID-19 data dashboard [8, 9].

### COVID-19 ward demand

In this step, we separated the weekly demand for general ward care by age into two tables; one for those cared for within the expected hospital capacity, and a second for those who fail to receive any hospital care when required because of lack of capacity.

### COVID-19 ICU demand

In this step we separated the weekly demand for ICU care by age into three tables; one for those cared for within the expected ICU capacity, a second for those who receive only ward care when ICU care is required because of no capacity, and a third for those who fail to receive any hospital care when required.

### Excess COVID-19 mortality sub-model

This is a critical step in the modelling as it is here that the estimate of the increase in COVID-19 mortality in the absence of a ward or ITU bed is determined. It would be very challenging to directly make a meaningful estimate of the increase in mortality overall, given the diverse range of severity of COVID-19 and the distribution of people with COVID-19 across the different pathways that unfold. In order to reduce uncertainty, it is necessary to map the flow of individuals through treatment pathways and consider the impact on mortality that would arise from depriving any individual requiring that element of care. The uncertainty in estimating the impact of lack of capacity is greatly reduced by considering each element of care in the pathway separately than when considering the impact across the pathway as a whole. Consequently, we have taken the approach of compartmentalizing the care-pathway for COVID-19, identifying the most critical element of the pathway for different individuals and the volume of people in those compartments from observed data. For example, for people who never need more than high-flow oxygen on a general ward (> 35% O2), they are allocated to this compartment and their risk is not related to lower-risk care compartments like ‘general ward care’. Similarly, for individuals who pass through general ward care and high flow oxygen to ITU admission, continuous positive airways pressure’ support (CPAP) and eventually invasive mechanical ventilation (IMV), then they are allocated to riskiest of these compartments—the IMV. In the latter case, we can observe the survival from data in this group and also make a reasonably reliable judgment that if the option of IMV is removed, then the survival will be close to zero. There will be greater uncertainty in the estimation of the impact of removing lower levels of care, but structure can be applied to estimating the impact of depriving this care by considering the fatal events that are averted by the care and their risk in the absence of that care. The evidence and assumptions used in setting the mortality rates in each compartment can be found in Table 2. All required judgements were made by CM, a former clinician and the principal architect of this sub-model in conjunction with RS, a physician in respiratory medicine and critical care.

**Table 1:** Hospital care compartments, proportion occupancy and the mortality rates under three capacity scenarios

Proportion	Care segment	Proportion in each group	COVID-19 Mortality: in capacity	COVID-19 Mortality: no ICU	COVID-19 Mortality: no ward or ICU
92%	General ward care	0.6	0.01	0.01	0.03
	Ward care: O_2_ > 35%	0.17	0.01	0.01	0.5
	Ward care: O_2_ > 35% (Ceiling)	0.23	0.4	0.4	0.95
8%	ICU: Supportive/HFO	0.48	0.01	0.02	0.75
	ICU: NIV/CPAP	0.16	0.01	0.02	0.95
	ICU: NIV/CPAP (Ceiling)	0.12	0.83	0.9	1
	ICU: IMV ± ECMO	0.24	0.4	1	1

ICU—intensive care unit, HFO—high flow oxygen, NIV—non-invasive ventilation, CPAP—continuous positive airways pressure, IMV—invasive mechanical ventilation, ECMO—extracorporeal membrane oxygenation, Ceiling—this refers to the most intensive level of care that is appropriate to each patient depending on their level of frailty and usually determined by the Clinical Frailty Score (CFS).

**Table 2:** Expert judgements made on mortality rates by care category in the excess mortality sub-model

	Assumption within capacity	Assumption with noICUbeds	Assumption with noICUor ward beds
General ward care	(1%) Very low mortality as these patients are un-escalatedand so will only occur with non-respiratory failure deaths or sudden deaths	(1%) Very low mortality as these patients are un-escalated andso will only occur with non-respiratory failuredeaths or sudden deaths	(3%) The risk remains low but will be greater than the mortality rate ward-based care
Ward care O_2_ > 35%	(1%) This is a group of people who would be escalated to ICUif they deteriorated, so will only include sudden deaths	(1%) Those who deteriorate may get CPAP on the ward, so the CPAP mortality rate from NIV/CPAP on ICU has been used	(50%) Assume substantial mortality as use ofO2 > 35% indicates severe disease and risk to life. Assume that there will be no O_2_ outside of hospital. Set at 50% after discussion with ICU consultant
Ward care O_2_ > 35% (Ceiling)	(40%) Assumption that this cohort will be frail with a CFS of 6–9. Mortality is about 40% at 28 days [11]	(40%)Assumption that this cohort will be frailwith a CFS of 6–9. Mortality is about 40% at 28 days [11]. Assume unaffected by ICU absence	(95%) This is a group of frail people who will have no medical support despite needing unusually high O_2_ flows to remain stable. Assume there will be no O_2 _outside of hospital
Supportive/HFO on ICU	(1%) Assume the same as for NIV/CPAP on ICU	(2%) Assume the same as for NIV/ CPAP on ICU	(75%) With an assumption that no O_2_ available outside hospital, mortality in this group is likely to be high without it. Identified at having a risk to life by their need for admission toICU
NIV/CPAP on ICU	(1%) There were 31 patients who received NIV only on ICU, none of whom died suggesting the mortality rate is below 3–4%. Deaths are only likely to occur if from non-respiratory causes such as sepsis or non-respiratory organ failure, otherwise they would have been escalated to IMV/ECMO [10]	(2%) Assume that CPAP therapy cancontinue on the ward, but mortality rate is double the 'within capacity' mortality rate because ofreduction in supportive care	(95%) Assume the vast majority would die without CPAP, otherwise they would have remained on high flow oxygen. Also assume that there will be no O_2_ outside hospital
NIV/CPAP on ICU(Ceiling)	(83%) 20 out of 24 patients who received NIV died. These patients were frail with a mean CFS of 6 and would not have been considered for escalation of care [10]	(90%) Assume that CPAP therapy cancontinue onthe ward, but mortality rate increasesdue to a reduction in supportive care	(100%) Assumption that in the absence of any hospital bed, there would be no NIV and no chance of survival
IMV ± ECMO on ICU	(40%) Since 1st September2020 mortality rate in those ventilated within 24hrs after admission is 40%. Assumption that the mortality rate is similar for those mechanically ventilated after24hrs [12]	(100%) It is a reasonably safe assumption that the mortality rate would be close to 100% as IMV/ECMO are dangerous but potentially lifesaving procedures; only carried out if considered life-saving in this circumstance	(100%) It is a reasonably safe assumption that the mortality rate would be close to 100% as IMV/ECMO are dangerous but potentially life-saving procedures; only carried out if consideredlife-saving in this circumstance

ICU—intensive care unit, HFO—high flow oxygen, NIV—non-invasive ventilation, CPAP—continuous positive airways pressure, IMV—invasive mechanical ventilation, ECMO—extracorporeal membrane oxygenation, CFS—clinical frailty score, Ceiling—this refers to the most intensive level of care that is appropriate to each patient depending on their level of frailty and usually determined by the Clinical Frailty Score (CFS).

We estimated the multiplier of COVID-19 mortality risk for ward and ICU care COVID-19 patients when there is (1) no ICU capacity and (2) when there is neither ward nor ICU capacity in comparison to the in-capacity mortality. The output from this sub-model is only dependent on the ‘hospitalised’ compartment and the modelling is not age dependent.

The proportion of patients requiring intensive care was taken from the results of the modelling step “Splitting Admissions into ICU and Ward” (8%). Those receiving general ward care or ICU care were segmented into the categories shown in Table 1. The distribution of ward care patients across the care categories was populated using observation data from the Nottingham Universities Hospitals Trust [10] The proportion of ICU patients only requiring supportive care or high flow oxygen was also take from the Nottingham data. The rest of the ICU categories were populated in line with published data from University Hospital Southampton for similar group of patients [11].

The expected numbers of deaths in each category calculated using the assumed mortality rate and weighted by the proportions in the categories using the formulae below.

$$q=\frac{\sum_{c=1}^{c=n}{deaths}_{c}}{hosp}$$

where $q=$ probability of dying in this episode of COVID-19. ${deaths}_{c}=$ the number of deaths in the care category ‘c’. $hosp=$ the number of people hospitalised with COVID-19

$$m=-(1-LN\left(1-q\right))$$

where $m=$ hazard rate for death corresponding to the probability of dying in the scenario ‘q’, and

$$HR=\frac{{m}_{s}}{{m}_{b}}$$

where $HR=$ hazard ratio applied to the base-case mortality rate ${m}_{b}$ to find the mortality rate in scenario ‘s’.

From the Excess Mortality Sub-model, we estimated a hazard ratio of 1.99 for COVID-19 mortality in those needing ICU care when no ICU bed was available but there was a ward bed, and 9.02 when there was neither an ICU nor ward bed available. For those needing ward care only, the hazard ratio is 3.69 when no hospital bed is available. Sensitivity testing indicated that results were not especially sensitive to small changes in these hazard ratios (± 20%).

### Ward mortality-treated

In this step, we estimated the in-capacity COVID-19 mortality rate by age in 5-year age bands interpolating from data from the cumulative COVID-19 daily deaths report on the NHS England website and using the age distributions for admissions calculated in the modelling step “Estimating Admissions by Age” [12]. Data was harvested from the 6th November 2020 dataset as this precedes the peak of the second wave when pressure may already have been building on internal hospital resources. After estimating the mid-points of the age-bands, an exponential model is fitted to the mortality rates for the three age bands from age 40 upwards. This fitted model is then used to interpolate the mortality rates for the 5-year age bands required.

### ICU mortality-treated

In this step, we calculated the in-capacity COVID-19 mortality rates by 5-year age bands in ICU. Data was taken from the 6^th^ November 2020 ICNARC report on COVID-19 in intensive care [13]. The mortality rate for each age band was calculated from the sum of the product of the number of admissions for that age band, the 28-day in-hospital mortality rate and the total number of admissions for that age band. An exponential model was then fitted to the data to which age bands had been applied in order to allow interpolation and re-categorising by 5-year age-bands.

### COVID-19 mortality rates

In this step we took the COVID-19 mortality rates by age calculated in the previous two modelling steps and the hazard ratios from the ‘Excess Mortality Model’ step to calculate the mortality rates when no ICU beds and no ward beds are available respectively. The mortality rate under the scenario ‘s’ is calculated using the equation:

$${q}_{s}=1-{\left(1-q\right)}^{{HR}_{s}}$$

where $q=$ the mortality rate in the in-capacity scenario. ${HR}_{s}=$ the hazard ratio in scenario ‘s’. ${q}_{s}=$ the mortality rate in the scenario ‘s’ (Table 2).

### COVID-19 deaths—in capacity

In this step, we calculated the number of COVID-19 deaths each week from the corresponding number of admissions generated by the ‘Ward Demand’ and ‘ICU Demand’ steps and the in-capacity mortality rates from the previous ‘Mortality Rates’ step. Observed data is used up to the 9^th^ December 2020, with ICU deaths calculated as 15% of the total number of deaths (as there are significant delays in reporting the deaths in the ICNARC data).

### Deaths—outside capacity

In this step, we calculated the numbers of deaths arising each week as a direct result of failing to get an ICU or ward bed. Observed data is used up to the 9th December 2020. A series of seven calculations were used:

1. The number of deaths in ICU within capacity as the dot product of the vector of ICU demand within ICU capacity by age from step 'ICU Demand' and the vector of mortality rates from step ‘Mortality Rates’.
2. The number of deaths in patients requiring ICU care where only ward care was available, as the dot product of the vector of ICU demand outside ICU by age from step 'ICU Demand' and the vector of mortality rates from step ‘Mortality Rates’.
3. The number of deaths in patients requiring ICU care where neither ward nor ICU care was available as the dot product of the vector of ICU demand outside both ICU and ward by age from step 'ICU Demand' and the vector of mortality rates from step ‘Mortality Rates’.
4. The number of deaths in patients requiring ward care only as the dot product of the vector of ward demand within ward capacity by age from step 'Ward Demand' and the vector of mortality rates from step ‘Mortality Rates’.
5. The number of deaths in patients requiring ward care where ward care was not available as the dot product of the vector of ward demand outside ward capacity by age from step 'Ward Demand' and the vector of mortality rates from step ‘Mortality Rates’.
6. The total number of deaths each week under the current scenario including both ICU and ward deaths as the sum of all results in steps 1–5.
7. The total number of deaths in each calendar week in both the ICU and ward care groups calculated in the step ‘Deaths—In capacity’ was taken and subtracted from the capacity constrained total in step 6 to provide the number of deaths arising as a direct result of the lack of an ICU or ward bed.

### Scenarios

We applied three scenarios for admission demand from the week beginning 16th December 2020 through to the week beginning 28th April 2021 (see Fig. 2).

In this model, the three scenarios are referred to as “Pessimistic”, “Middling” and “Optimistic” and were arbitrary but based on the trajectory of the increase in the multiplier of admissions from one week to the next. The trajectory was calculated as a linear regression of the last three weeks of the known data taken from the GOV.UK Coronavirus dashboard [8]. They are illustrative scenarios and not forecasts. With the passage of time, further data through to the week of the 3rd of February was obtained for comparison with the scenarios. The peak in admissions actually occurred around the week of the 13th of January.

In the Middling scenario, the multiplier continued to increase following the rate over the previous 3 weeks up to three weeks post the introduction of more vigorous countermeasures on 19^th^ December 2020. This is to be expected as the incubation period is nearly a week, so the enhanced countermeasures would usually take at least a week to result in any observed change. The multiplier falls below 1.0 after 4 more weeks. The emergence of a new variant of SARS-CoV-2 with higher transmission rates that became more prevalent from the beginning of December has driven more rapid growth of infection rates. As the new variant makes up a greater proportion of new cases, the rate of growth will continue to rise unless effectively mitigated. It remains uncertain, at the time of writing, how it will respond to tighter controls, but there is no reason to assume the mortality rates will differ at present. This new variant has only recently been identified and has not been factored into the analysis. However, initial indications are that this variant could open up a variety of more pessimistic scenarios in the short term.

We now have effective vaccines and it is expected that their use will prevent a large number of infections and many deaths [14].

In the Optimistic scenario, the multiplier begins to decelerate from week beginning 16th December, continues to decelerate after the 16th December and falls below 1.0 after 3 weeks.

In the Pessimistic scenario, the multiplier continued to increase for 4 weeks after week beginning 16th December before decelerating at the same rate as the other two scenarios. This is not intended as a worst-case scenario, but one that is somewhat worse than the ‘Middling’ scenario.

### Sensitivity analysis

We performed an analysis to assess the sensitivity of the model to the choice of capacity and hazard ratio parameter values and scenario. Five input parameters were varied independently in turn and the effect on the model output recorded. The five parameters varied were the ICU and ward capacities and the hazard ratios for mortality in ward patients when there are no ward beds available and ICU patients when there are no ICU beds and ICU patients when there are no ward beds available. The hazard ratios were adjusted using the equation.

$${HR}^{*}=1+\left(HR-1\right).(1+d)$$

where $HR=$ the within-capacity hazard ratio. ${HR}^{*}=$ the outside-capacity hazard ratio. $d=$ the adjustment to be applied as a proportion.

This model has been populated with data specific to England, it could be applied in other geographies and we have added ways of estimating some data like the age distribution of admissions or the ICU admission rates by age. Where this data is directly available it can be substituted.

### Assumptions

There are several general underlying assumptions to the model.

- There is no change in the expected mortality as demand grows until the bed capacity is breached.
- Only bed capacity affects excess mortality rates and not other resource constraints such as staffing, equipment, ambulance availability or other finite resources, although actual system capacity may be a product of all of the above.
- Health care policy and conscious or unconscious clinician behaviour that may affect the compartments of care through which the patients flow does not change as the pandemic waxes and wanes.
- Surge capacity will be applied before the use of field hospitals or the commandeering of private facilities.
- All the surge capacity, field hospital and private facility beds will be utilised for COVID-19 rather than any other demand.
- The age distribution of admissions remains constant over time.
- There is perfect redistribution of bed capacity and resources across the country immediately according to demand.
- Weekly bed availability remains constant.
- Capacity estimates are fixed throughout the model when, in fact, new resources may be recruited over time, or capacity may fall in response to disruption of staffing and supplies.
- The mortality rates, including by age, are the same for the old COVID strain and novel mutations.

## Results

The key output of our collaboration was the model itself rather than the results of any of the scenarios. The model allows a user to understand the excess COVID-19 mortality impact arising as a direct consequence of ward and/or ICU capacity being breached under various scenarios or forecasts of hospital admissions. The scenarios described in this paper are illustrative and are not forecasts (Table 3).

**Table 3:** Numbers of deaths with the unadjusted middling scenario from 16th of December (bold font)

Week	Total COVID-19 Deaths (with ICU and Ward capacity limits)	COVID-19 deaths if there are no ICU and Ward capacity limits	Excess COVID-19 deaths specifically due to ICU and Ward capacity limits
30/09/2020	298	298	0
07/10/2020	444	444	0
14/10/2020	731	731	0
21/10/2020	1074	1074	0
28/10/2020	1457	1457	0
04/11/2020	1784	1784	0
11/11/2020	1897	1897	0
18/11/2020	2103	2103	0
25/11/2020	2031	2031	0
02/12/2020	1889	1889	0
09/12/2020	1960	1960	0
**16/12/2020**	2685	2685	**0**
**23/12/2020**	3703	3703	**0**
**30/12/2020**	5697	5697	**0**
**06/01/2021**	9677	9677	**0**
**13/01/2021**	11,689	11,612	**77**
**20/01/2021**	11,689	11,612	**77**
**27/01/2021**	9289	9289	**0**
**03/02/2021**	7432	7432	**0**
**10/02/2021**	5945	5945	**0**
**17/02/2021**	4756	4756	**0**
**24/02/2021**	3805	3805	**0**
**03/03/2021**	3044	3044	**0**
**10/03/2021**	2435	2435	**0**
**17/03/2021**	1705	1705	**0**
**24/03/2021**	1193	1193	**0**
**31/03/2021**	835	835	**0**
**07/04/2021**	585	585	**0**
**14/04/2021**	409	409	**0**
**21/04/2021**	286	286	**0**
**28/04/2021**	201	201	**0**
**Total**	**102,728**	**102,573**	**154**

### Sensitivity analyses

**Fig. 3:**
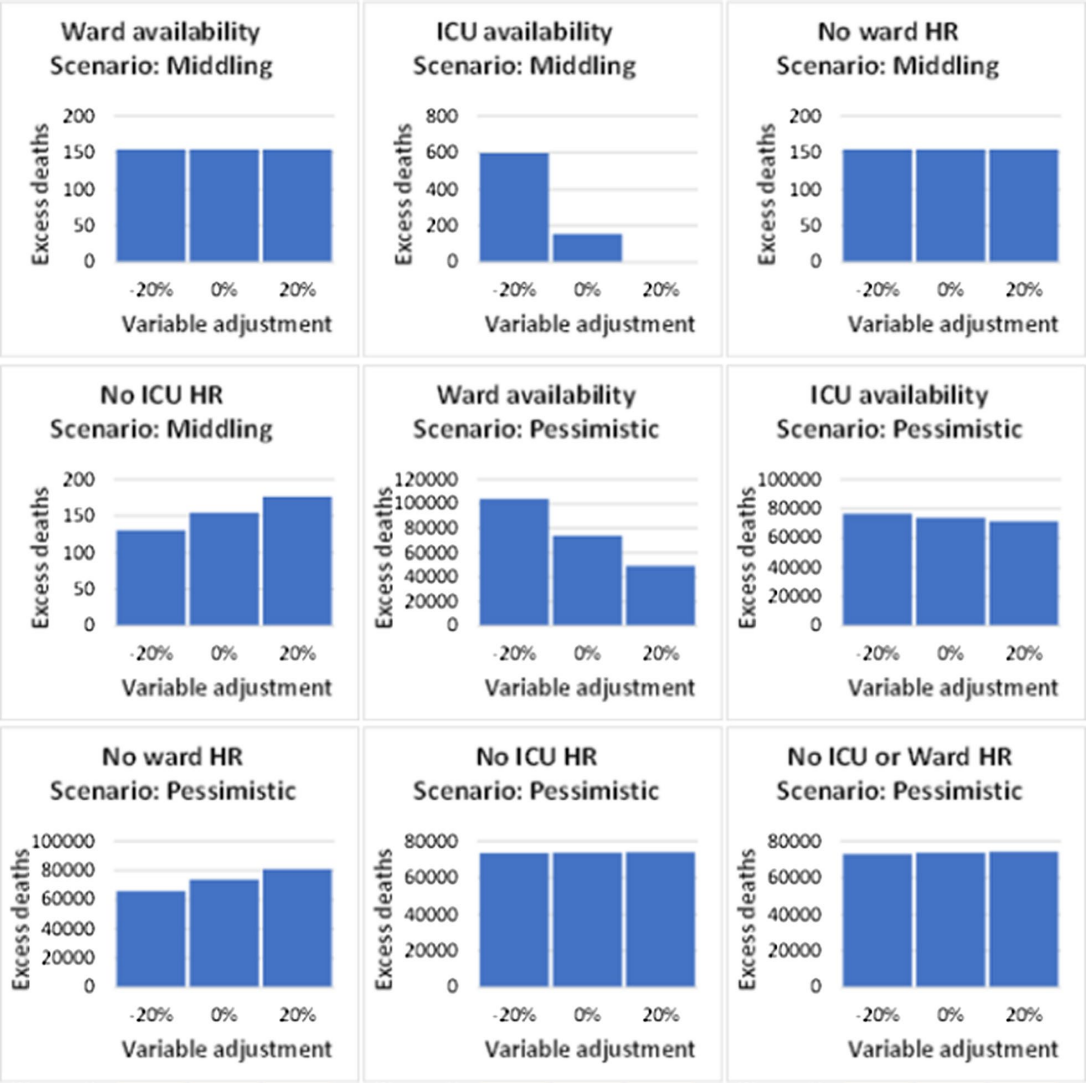
Results of the sensitivity analysis

The results of the sensitivity analysis are shown in Table 4 and Fig. 3. None of the Optimistic scenario adjustments resulted in any excess deaths. In the Middling scenario adjustments, the excess deaths attributable to lack of capacity alone ranged from 0 to 597 with a 20% reduction in the expected number of ICU beds. In the Pessimistic scenario the number of excess deaths attributable directly to lack of capacity range from 49,178 with a 20% increase in the number of ward beds, and 103,735 with a 20% reduction in the expected ward bed availability.

**Table 4:** Results of the sensitivity analysis

Scenario	Ward availability (%)	ICU availability (%)	No ward HR (%)	No ICU HR (%)	No ICU or Ward HR (%)	Excess COVID-19 deaths due to capacity breach	Total COVID-19 deaths	% increase in COVID-19 deaths
Middling	− 20	0	0	0	0	102,573	154	102,728
Middling	0	0	0	0	0	102,573	154	102,728
Middling	20	0	0	0	0	102,573	154	102,728
Middling	0	− 20	0	0	0	102,573	597	103,170
Middling	0	20	0	0	0	102,573	0	102,573
Middling	0	0	− 20	0	0	102,573	154	102,728
Middling	0	0	20	0	0	102,573	154	102,728
Middling	0	0	0	− 20	0	102,573	130	102,703
Middling	0	0	0	20	0	102,573	176	102,750
Middling	0	0	0	0	− 20	102,573	154	102,728
Middling	0	0	0	0	20	102,573	154	102,728
Optimistic	0	0	0	0	0	47,073	0	47,073
Pessimistic	− 20	0	0	0	0	289,983	103,735	393,718
Pessimistic	0	0	0	0	0	289,983	73,711	363,694
Pessimistic	20	0	0	0	0	289,983	49,178	339,161
Pessimistic	0	− 20	0	0	0	289,983	76,382	366,365
Pessimistic	0	20	0	0	0	289,983	71,253	361,236
Pessimistic	0	0	− 20	0	0	289,983	66,576	356,559
Pessimistic	0	0	20	0	0	289,983	79,764	369,747
Pessimistic	0	0	0	− 20	0	289,983	73,472	363,455
Pessimistic	0	0	0	20	0	289,983	73,926	363,909
Pessimistic	0	0	0	0	− 20	289,983	72,943	362,926
Pessimistic	0	0	0	0	20	289,983	74,197	364,180

The results can be explained by considering how capacity evolves in each of the scenarios. In the Middling scenario, whilst ICU capacity may be approached and even possibly breached, there remains sufficient ward capacity to take lives who need either ward or ICU support, keeping excess deaths relatively low. However, the Pessimistic scenario sees ward capacity breached, and in many scenarios for a period of several weeks, resulting in much higher mortality in those lives who require care but do not receive it. ICU capacity is much lower than ward capacity and only a small proportion of all hospitalized patients need ICU care so the number of deaths from breaches of ward capacity are proportionally larger than breaches for ICU care. ICU care is assumed to reach capacity before ward care, so with marginal breaches of capacity, ICU breaches are the source of the excess deaths, but with large breaches of capacity, the majority will arise from breaching of ward capacity.

The number of excess deaths is most sensitive to ward bed availability in the pessimistic scenario with a difference of 54,556 excess deaths between the 20% increase and 20% decrease in the bed availability compared to 5,129 with the same variation in ICU bed availability. However, in the Middling scenario, the excess deaths are most sensitive to ICU bed capacity with a difference of 597 with ± 20% variation in the ICU bed capacity estimate, with no difference arising from the ward bed capacity estimate.

These results are shown graphically in Fig. 2 (note the different y-axis scales). Excess deaths are more sensitive to the availability of ICU or ward beds than to the adjustments in the hazard ratios used here. The greatest loss of life occurs with a 20% reduction in the estimate of ward availability in the pessimistic scenario with 103,735 (35.8% increase) excess deaths.

The actual observations including data from the 16th December through to the week of the 3rd of February showed a peak weekly admission rate of 29,447 in the week of the 13th of January before declining (Fig. 2). There would have been no excess deaths due to lack of capacity in the observed cases through to February under this set of assumptions.

## Discussion

The new variant of SARS-CoV-2 that emerged in the UK (B.1.1.7) appears to be about 56% (95% CI 50%-74%) more transmissible than the existing variants and appears to have a higher case-fatality rate [15, 16]. Alternative explanations for its rise in prevalence and the increased rate of transmission observed since its appearance at the beginning of October were investigated including “immune escape” where individuals previously infected return to susceptibility as a result of mutation of key antigens, increased susceptibility amongst children, and a shorter generation time. None of these alternative explanations fitted the data as well as increased infectiousness. In addition, a variant has emerged from South Africa (501Y.V2) which also appears to have greater transmissibility and an increased viral load [17]. These unsettling developments increases uncertainty in the future trajectory of hospital demand and open up a variety of significantly more pessimistic scenarios in the short term that have not been possible to explore here.

In the longer term, there are now three vaccines approved for use in the UK, as of the 19th January 2021 [18]. It is expected they will have a substantial impact on admissions and deaths, though not until after the majority of deaths in our scenarios are modelled as occurring [19]. Consequently, we have not factored the application of vaccination into the scenarios as they are operating in the short-term.

## Limitations

Here we list and discuss the limitations of the model:

- The estimate of bed availability is determined using the mean length of stay on the ward or in ICU. The distribution of occupants by length of stay will change over time which may result in a slow consumption of capacity that is not captured in this model. For example, a higher proportion of afflicted younger patients in hospital may lead to longer bed occupancy both in ward and critical care, as withdrawal of active treatment in view of futility is less likely to take place in this group.
- Mortality rates are affected by constraints other than just bed availability including staffing and equipment.
- The model will not capture the transition between low mortality with full capacity and the high mortality from lack of a bed that arises from stressing of the system before capacity is absent [20].
- Capacity estimates are fixed throughout the model when, in fact, new resources may be recruited over time, or capacity may fall in response to disruption of staffing and supplies.
- Age specific case fatality rates are assumed to be static, but in fact may change, either due to changes in virulence, improvement in care or dilution of standards of care during a surge scenario.
- The oxygen cut-off of 35% is based on clinical opinion, and there is no empirical study in support of this threshold for obvious reasons. However, often clinical practice may be to err on the side of caution in a ward setting and to keep a patient on a slightly higher fractionated oxygen than s/he needs and therefore a patient on a lower than 35% FiO2 may actually need even smaller amounts of oxygen, and therefore the assumption is that s/he would withstand the lack of medical oxygen with a degree of success.

Intensive care resources are constrained not only by bed availability but also by equipment and staffing. A combination of all three is required for optimal care in ICU. In reality there is not a simple binary state of presence or absence of these factors and skills. A degradation of equipment maintenance, training around new equipment and distribution as well as reductions in the effectiveness of staff, either because staff-to-patient ratios fall as demand rises, or because of staff sickness due to COVID-19 or simply the physical and emotional fatigue as the pandemic continues is also seen. An analysis of within-capacity mortality rates in ICU by bed occupancy has found that there is an almost linear increase in mortality with no excess mortality at 0% bed occupancy to a 92% increase in mortality by 100% occupancy [20].

The modelling by McCabe et al. suggests that ICU capacity is first constrained by bed availability, though lack of nurses and junior doctors is close behind [2]. They included sickness absence rates taken from surveys of union members suggesting 15% of doctors were off sick in the first wave and may under-estimate the impact of sickness on staffing overall at peak times in the pandemic [21]. As the pandemic progresses, higher than anticipated absence due to sickness in these groups could result in lack of limiting capacity before the lack of beds, both in ICU and in wards. Reorganisation within hospitals may mitigate this by training other staff to support ICU work and thereby increasing ICU staff to bed ratios [22]. One hospital managed to meet demand in the first wave, but reduced ICU nurse to bed ratios from the normal pre-pandemic of 1:1 to 1:4 [4]. Current guidance on nursing staff ratios during the COVID-19 pandemic advocates a ratio of 1:2 [22].

Decision making may change in the face of increasing demand either with formal revisions of treatment thresholds as resources become increasingly scarce or with the introduction of triaging. On a less formal level, the heuristics used by clinicians in their everyday management of patients may vary as competing pressures rise. For example, at times of abundant capacity, the thresholds for transferring patients into ICU for a trial to see if a patient with poor chance of survival recovers with a short stay, may be lower than when there is severe limitation on capacity.

One of the most uncertain parts of the modelling is the determination of the multipliers of risk in the section ‘Excess Mortality Submodel’. We estimated that the multiplier for the scenario of no ICU bed when one was required was 1.99. A recent observational study of 4,032 ICU admissions across 114 hospital trusts found a rising mortality with bed occupancy [20]. The estimated excess mortality rate was estimated to reach 92% at 100% capacity. This is very close to our estimate of 99% and is a reassuring triangulation of the method.

An important but unrealistic assumption in the model is that there is perfect distribution of resources with respect to demand across the country and that no patient is refused care whilst there remains a bed anywhere in England. ICUs are organized into networks which facilitate the transfer of patients from one hospital to another when ICU beds in a hospital run out, but even a delay of a few hours in transferring a patient to an ICU bed can influence outcome. Furthermore, even in the case of bed availability in a different hospital, it cannot be assumed that the patient in the referring and at-capacity hospital may be fit enough to able to be transferred safely. All the ICUs in a network are likely to have correlated demand and may all run out of space at the same time. In these circumstances, transfers would need to be arranged between networks or regions and this would entail yet further delay and challenges with consequent impact on outcomes.

It is notable that the sensitivity of the excess deaths to ICU capacity is much greater than it is to ward bed capacity at marginal breaches of capacity, but that large scale breaches of capacity are more sensitive to ward bed capacity. There are far more ward beds than ICU beds, and there is an assumption that ICU capacity would be exhausted long before ward bed capacity.

## Conclusions

Here we describe a demand and capacity model for general hospital and intensive care beds in the context of the COVID-19 pandemic in England. The model allows a user to understand the excess COVID-19 mortality impact arising as a direct consequence of capacity being breached under various scenarios or forecasts of hospital admissions. The scenarios described in this paper are illustrative and are not forecasts.

We estimated the number of excess COVID-19 deaths up to the end of April 2021 that would arise from lack of ward and ICU capacity under different demand assumptions from December 16th, 2020. No excess deaths from excess capacity would be expected under the ‘Optimistic’ assumptions of demand but would reach between 49,178 and 103,735 under the ‘Pessimistic’ scenario. Without the new variant, exceeding capacity for hospital and ICU beds was not the most likely outcome but given the new variant it appeared more plausible and could have result in a substantial increase in the number of COVID-19 deaths. In the event, it would appear that capacity was not breached at a national level any stage, and, under an assumption of a perfectly even distribution of demand and capacity, there would be no excess deaths due to lack of capacity expected under this set of assumptions. However, distribution of demand and capacity is imperfect. It will remain unclear if minor local capacity breaches resulted in any small number of excess deaths.

## Supplementary Information

**Additional file 1**. https://github.com/Crystallize/COVID19_ExceedingCapacityModel. Link to model on GitHub.

## Data Availability

The dataset supporting the conclusions of this article is available GitHub: https://github.com/Crystallize/COVID19_ExceedingCapacityModel..

## Abbreviations

CFR: Case fatality rate
CFS: Clinical frailty score
CPAP: Continuous positive airway pressure
ECMO: Extracorporeal membrane oxygenation
HFO: High flow oxygen
HR: Hazard ratio
ICNARC: Intensive care national audit and research centre
ICU: Intensive care unit
IFR: Infection fatality rate
IMV: Invasive mechanical ventilation
NIV: Non-invasive ventilation
ONS: Office of national statistics
SARS-CoV-2: Severe acute respiratory syndrome coronavirus 2
VOC: Variant of concern
